# Molecular mapping of quantitative trait loci for 3 husk traits using genotyping by sequencing in maize (*Zea mays* L.)

**DOI:** 10.1093/g3journal/jkac198

**Published:** 2022-08-09

**Authors:** Jun Zhang, Fengqi Zhang, Lei Tian, Yong Ding, Jianshuang Qi, Hongfeng Zhang, Xinyuan Mu, Zhiyan Ma, Laikun Xia, Baojun Tang

**Affiliations:** Cereal Crops Research Institute, Henan Academy of Agricultural Sciences, Henan Provincial Key Laboratory of Maize Biology, Zhengzhou 450003, China; Cereal Crops Research Institute, Henan Academy of Agricultural Sciences, Henan Provincial Key Laboratory of Maize Biology, Zhengzhou 450003, China; Henan Institute of Science and Technology for Development, Zhengzhou 450003, China; College of Agronomy, State Key Laboratory of Wheat and Maize Crop Science, Center for Crop Genome Engineering, Henan Agricultural University, Zhengzhou 450046, China; Cereal Crops Research Institute, Henan Academy of Agricultural Sciences, Henan Provincial Key Laboratory of Maize Biology, Zhengzhou 450003, China; Cereal Crops Research Institute, Henan Academy of Agricultural Sciences, Henan Provincial Key Laboratory of Maize Biology, Zhengzhou 450003, China; Henan Institute of Science and Technology for Development, Zhengzhou 450003, China; Cereal Crops Research Institute, Henan Academy of Agricultural Sciences, Henan Provincial Key Laboratory of Maize Biology, Zhengzhou 450003, China; Cereal Crops Research Institute, Henan Academy of Agricultural Sciences, Henan Provincial Key Laboratory of Maize Biology, Zhengzhou 450003, China; Cereal Crops Research Institute, Henan Academy of Agricultural Sciences, Henan Provincial Key Laboratory of Maize Biology, Zhengzhou 450003, China; Cereal Crops Research Institute, Henan Academy of Agricultural Sciences, Henan Provincial Key Laboratory of Maize Biology, Zhengzhou 450003, China

**Keywords:** maize, husk traits, genotyping by sequencing, quantitative trait locus, single-nucleotide polymorphism

## Abstract

The maize (*Zea mays* L.) husk consists of multiple leaf layers and plays an important role in grain growth and development. Despite significant achievements in physiological and morphological research, few studies have focused on the detection of genetic loci underlying husk-related traits due to the lack of efficient tools. In this study, we constructed an ultra-high-density linkage map using genotyping by sequencing based on a recombinant inbred line population to estimate the genetic variance and heritability of 3 husk traits, i.e. husk length, husk width, and husk layer number in 3 field environments and the combined environment. The 3 husk traits showed broad phenotypic variation and high heritability; the broad-sense heritability (*H*^2^) was 0.92, 0.84, and 0.86. Twenty quantitative trait loci were consistently detected more than 1 environment, including 9 for husk length, 6 for husk width, and 5 for husk layer number. These loci were considered as stable quantitative trait loci. Based on the quantitative trait loci mapping in the recombinant inbred line population, q*HL*6 and q*HN*4 were detected across all environments and inferred to be reliable and major-effect quantitative trait loci for husk length and husk layer number, respectively. In addition, several predicted candidate genes were identified in the region of q*HL*6 and q*HN*4, of which 17 candidate genes potentially play a role in biological processes related to development process and energy metabolism. These results will be as a useful resource for performing functional studies aimed at understanding the molecular pathways involved in husk growth and development.

## Introduction

Maize (*Zea mays* L.) is one of the most important cereal and forage crops grown worldwide (Cui *et al.* 2018). Maize husk, a leaf-like tissue covering the outside of a maize ear, consists of multiple leafy layers, and it has important functions. The husk can provide a good environment for the development of kernels and protect them from birds, pests, and pathogen infections (Wang *et al.* 2012; Cao *et al.* 2014; Cui *et al.* 2020). The husk is also a temporary storage for nutrients from other organs; it participates in photosynthesis to provide energy for kernel development, and it directly or indirectly provides plentiful sources of anthocyanins and fiber for industrial production (Li *et al.* 2008). Furthermore, the husk has a higher conversion efficiency for photosynthetic products than other leaves under the same area, and it significantly contributes to the development of the ears (Cantrell and Geadelmann 1981; Fujita *et al.* 1995). Despite the importance of maize husk for crop yield and industrial production, there is limited information on its genetic and molecular mechanisms that govern husk architecture.

The husk architecture has several main traits such as husk layer number (HN), husk length (HL), and husk width (HW) that influence the level of husk function. A fairly close relationship exists between husk phenotypes and corresponding features of the ear (Cui *et al.* 2016). The moisture content of the maize kernel at the harvest stage has shown significant positive correlations with HN and HL (Zhou, Hao, *et al.* 2016). When compared with leaves that initiate from the shoot apical meristem, these husk traits are affected by initiation and elongation of the lateral meristem, which involves cell division, differentiation, and metabolism (Avramova *et al.* 2015). Despite significant achievements in physiological and morphological research, the genetic and molecular mechanisms underlying husk-related traits are still largely unknown.

To date, several quantitative trait loci (QTLs) and genes have been found to regulate the development of husk architectural traits. For example, Zhou, Hao, *et al.* (2016) identified 8 and 9 stable single-nucleotide polymorphisms (SNPs) for husk number and weight, respectively, in a genome-wide association study (GWAS). In addition, Cui *et al.* (2016) identified 9 SNPs significantly associated with 4 husk traits in a GWAS and then performed a combination of linkage analysis and GWAS to predict 5 candidate genes for husk traits (Cui *et al.* 2018). Furthermore, Cui *et al.* (2020) used a recently developed GWAS method (BLINK) to identify markers associated with husk traits and revealed 6 genetic loci associated with HN and husk thickness above the Bonferroni multiple-test threshold. Zhou *et al.* (2020) fine-mapped a major-effect QTL (q*HN7*) for husk number and predicted 4 genes associated with plant growth and development. However, given that the lack of fundamental knowledge on the genetic basis underlying husk architecture, more QTL and candidate genes involved in the regulation of husk development need to be identified.

Linkage maps have been constructed for maize husk traits using a range of molecular markers including amplified fragment length polymorphisms, simple sequence repeats (Zhou, Hao, *et al.* 2016; Cui *et al.* 2016), and genome resequencing (Cui *et al.* 2018, 2020). Genotyping by sequencing (GBS) has recently been the method of choice for building linkage maps in maize to allow more complex traits to be mapped and selected for using marker-assisted selection in the future (Romay *et al.* 2013; Wang, Yuan, *et al.* 2020). Thus, to understand the genetic basis and regulatory mechanisms responsible for the husk traits, we constructed an ultra-high-density linkage map by GBS method from a recombinant inbred line (RIL) population whose parents exhibit significant differences in husk traits to estimate the genetic variance and heritability of 3 husk traits, i.e. HL, HW, and HN, in both 3 field environments and combined environment. We identified stably expressed QTLs that significantly affect the 3 husk traits. Furthermore, we predicated candidate genes involved in maize husk growth and development. The findings of this study will help us to understand the genetic basis of and molecular mechanisms that govern husk development.

## Materials and methods

### Plant materials and field experiments

An RIL population composed of 310 lines, developed from a cross between maize inbred lines PD80 and PHJ65, was used for the QTL mapping population. The parents have been publicly released by the national technical system of the maize industry. PD80 with slow field grain drying rate exhibits more HN, longer HL, and wider HW than related traits of PHJ65 with fast grain dehydration rate (Zhang *et al.* 2020). In 2020, the RIL population and parents were planted at 3 field environments in China: Zhoukou (E1, 108°E, 18°N), Xinxiang (E2, 113°E, 35°N), and Anyang (E3, 114°E, 36°N). Each line was grown in double rows (3.0 m in length with 0.6 m between rows), at a planting density of 65,000 plants ha^−1^, following a randomized complete block design with 2 replications per field environment. The plants, along with the guard rows, were under standard irrigation and fertilization management throughout the developmental period. Agronomic management of the field experiments was identical in each field environment. All plants were grown under open-pollination conditions.

### Phenotyping and statistical analyses

HN was counted from the outermost to innermost layers of the husk. HL was defined as the longest layer of the husk from the tip to the base. HW was determined by measuring the middle section of the third layer of the husk (Cui *et al.* 2016), which is more representative and stable for studying the husk-related trait, and it is not easy to be affected by the external environment, such as pests and diseases. The 3 husk traits of each line were measured from 6 well-pollinated plants grown in the same block at the maturity stage. The mean of these 6 individual trait values was calculated as the trait value for each line in per block. Then, the mean values from 2 block replications was taken as the phenotypic value of each field environment.

The statistical analysis was performed using R 3.1.1 (https://www.R-project.org/). Analysis of variance of HN, HL, and HW was performed using the *lmer* function of the lme4 package of R (Bates *et al.* 2014) based on the following model: $y_{\mathrm{ijk}}=\mu +{\text{env}}_{i}+{\mathrm{rep}(\mathrm{env})}_{ij}+{\text{genotype}}_{k}+\mathrm{env}\times {\mathrm{genotype}}_{ik}+\varepsilon_{\mathrm{ijk}}$, where *μ* is the grand mean of husk traits, ${\text{env}}_{i}$ is the environmental effect of the *i*th environment, ${\mathrm{rep}(\mathrm{env})}_{ij}$ is the effect of the *j*th replication within the *i*th environment, ${\text{genotype}}_{k}$ is the genotypic effect of the kth line, $\mathrm{env}\times {\mathrm{genotype}}_{ik}$ is the effect of interaction between the environmental and genetic effects, and $\varepsilon_{\mathrm{ijk}}$ is the residual error containing all the experimental factors above. All terms were fitted as random effects with the exception of μ. All of the variance components in the mixed model were to calculate the broad-sense heritability $H^{2}$ for the 3 husk traits: $H^{2}(\%)=\sigma_{g}^{2}/(\sigma_{g}^{2}+\sigma_{ge}^{2}/n+\sigma_{e}^{2}/nr)\times 100\%$, where $\sigma_{g}^{2}$, $\sigma_{ge}^{2}$, and $\sigma_{e}^{2}$ represent the genotypic variance, the variance of the interaction of genotype with environment, and the residual error, respectively, whereas n is the number of environments, and r is the number of replications (Hallauer *et al.* 2010). The descriptive statistics for the RIL population and parents were measured using SPSS 22.0 (SPSS, Chicago, IL, USA). The R software was used to analyze the correlation between various traits. To minimize the effects of the environment, the best linear unbiased predictor (BLUP) for the 3 husk traits across the 3 field environments was estimated using the same package of R. For each trait, the phenotype data from 6 block in 3 field environments was used to calculate the BLUP as combined environment. All phenotype data from each field environment and BLUP were used for subsequent analyses.

### Library preparation and sequencing

The genomic DNA of the 2 parents and RIL plants was extracted from fresh leaf tissue by using the cetyltrimethyl ammonium bromide method with minor modifications (Liu *et al.* 2003). GBS Library construction and sample indexing were performed as described previously (Elshire *et al.* 2011) and, then, developed using the ApeKI restriction enzyme and set of 96 barcodes. For each sampling, a single individual was used for genome sequencing on the Illumina HiSeq PE150 platform. Reads with ≥10% unidentified nucleotides and >50% bases with Phred quality <5 were filtered before alignment. Paired-end reads were mapped to the maize DNA reference genome with the Burrows-Wheeler Aligner (Li and Durbin 2009). Only the reads mapped uniquely to the reference genome sequence were used to call SNPs.

### Genotyping and construction of genetic linkage maps

SNP identification was performed using TASSEL 3.0 GBS Discovery Pipeline with B73 as the reference genome (http://www.maizegenetics.net/tassel/docs/TasselPipelineGBS.pdf). Bins serving as genetic markers were used for the construction of the genetic linkage map with JoinMap version 4.0, recombination frequency <0.4, and minimum logarithm of odds (LOD) score of 6 (Van Ooijen 2006), followed by a chi-square test to exclude the markers with segregation distortion. The Kosambi mapping function was used to calculate the genetic distance between markers (Kosambi 1943). QTL mapping in a single environment was performed with windows QTL cartographer 2.5 and the composite interval mapping (CIM) method (Basten *et al.* 1997). The entire genome was scanned every 0.5 cM, with a window size of 10 cM. Model 6 of the Zampqtl module was selected to detect QTLs and their effects. Forward–backward stepwise regression with 5 controlling markers was used to control the background from flanking markers. The confidence interval of QTL positions was estimated with the 1.5-LOD support interval method. To detect QTL × environmental interaction effects, joint mapping analysis was performed using ICIM-ADD of the MET functional module in QTL IciMapping v4.1 software (Meng *et al.* 2015). The threshold LOD value was determined empirically at a significance level of *P* < 0.05 by 1,000 permutations. Any QTL with an explained phenotypic variation (*R*^2^) of >10% was defined as a major QTL.

### Candidate gene analysis

According to the physical position of the SNP markers on both sides of each QTL position, a QTL physical interval was set up, and the genes in these intervals were predicted using the Gramene database (http://www.gramene.org/). The sequences in these intervals were then aligned in the MaizeGDB database to find the matching EST sequence and annotated the genes. Next, the biological function of these predicted gene was predicated in agriGO website (http://systemsbiology.cau.edu.cn/agriGOv2/).

## Results

### Phenotypic analysis

In this study, *t*-tests were performed to determine whether there were any significant differences in these husk traits (HL, HW, and HN) between the parents of RIL population. Significant differences were detected between the parents in 3 field environments (Table 1). Based on the BLUP values, HL was ∼15% shorter, HW was ∼29% narrower, and HN was ∼38% lower in P2 (HL was 17.70 cm, HW was 5.75 cm, and HN was 6.46 cm) compared with those of P1 (HL was 20.71 cm, HW was 8.02 cm, and HN was 10.28). In addition, broad phenotypic variation was observed in the RIL population, ranging from 6.27 to 15.80 cm in HN, 4.99 to 10.85 cm in HW, and 14.39 to 24.81 cm in HL. Analyses of the BLUP values showed that the mean values of the husk traits in the RIL population were close to the mid-parent values. The variances of genotype, environment, and genotype × environment (G × E) interactions were significant (*P < *0.01) for HL, HW, and HN, repetition within the environment (env/rep) was no-significant (Table 2). Analysis of variance revealed that there was statistically significant variance effect of genotype and environment on these husk traits in 3 field environments. Broad-sense heritability (*H*^2^) estimates were calculated, and the results revealed moderate heritability for all 3 husk traits, HL (*H*^2^ = 0.92), HW (*H*^2^ = 0.84), and HN (*H*^2^ = 0.86), indicating that it is largely determined by genotype (Table 2). Furthermore, HL, HW, and HN approximately fitted normal distributions with little skewness and kurtosis, except for HN in Yuanyang (Supplementary Fig. 1). These results indicate that most of the phenotypic variations in husk phenotypes are controlled by genetic factors and are suitable for further QTL mapping.

**Table 1.** Summary of the trait data for the RIL mapping population compared to parental performance in 3 field environments and combined environment.

Trait^a^	Environment^b^	Parents			RILs			
		P_1_ ± SD	P_2_ ± SD	*P-*value*^c^*	Mean ± SD	Range	Skewness	Kurtosis
HL	E1 (Zhoukou)	20.63 ± 0.41	17.66 ± 0.29	0.004	18.89 ± 0.12	13.30–24.40	0.04	−0.13
	E2 (Yuanyang)	19.97 ± 0.33	17.08 ± 0.13	0.010	19.61 ± 0.12	14.00–25.70	0.07	−0.07
	E2 (Anyang)	21.52 ± 0.29	18.36 ± 0.68	0.008	19.74 ± 0.14	13.94–25.72	0.04	−0.07
	Combined (BLUP)	20.71 ± 0.34	17.70 ± 0.37		19.43 ± 0.11	14.39–24.81	0.03	−0.09
HW	E1 (Zhoukou)	8.26 ± 0.21	5.56 ± 0.24	0.000	7.43 ± 0.07	4.60–11.14	0.31	0.35
	E2 (Yuanyang)	7.59 ± 0.28	5.92 ± 0.41	0.000	7.14 ± 0.06	4.50–10.84	0.22	−0.17
	E3 (Anyang)	8.22 ± 0.37	5.76 ± 0.30	0.000	8.26 ± 0.06	5.86–11.28	0.29	−0.16
	Combined (BLUP)	8.02 ± 0.29	5.75 ± 0.32		7.61 ± 0.32	4.99–10.85	0.32	0.06
HN	E1 (Zhoukou)	11.80 ± 0.40	6.36 ± 0.25	0.000	9.40 ± 0.08	6.40–15.00	0.5	0.24
	E2 (Yuanyang)	11.93 ± 0.38	6.60 ± 0.24	0.000	8.99 ± 0.09	6.00–15.80	0.87	−0.89
	E3 (Anyang)	10.10 ± 0.46	6.42 ± 0.32	0.002	9.42 ± 0.09	6.00–16.60	0.67	0.75
	Combined (BLUP)	10.28 ± 0.41	6.46 ± 0.27		9.27 ± 0.08	6.27–15.80	0.71	0.95

SD, standard deviation.

a Trait is the name of the component of husk: HL, HW, and HN.

b Environment: E1 is Zhoukou; E2 is Yuanyang; E3 is Anyang; and combined is the best linear unbiased predictions values (BLUP) for each trait of each line across 3 field environments.

c *P*-value based on a *t*-test evaluating 2 parental lines.

**Table 2.** Variance components estimate, significance tests, and broad-sense heritability of 3 husk traits in RIL population.

Variance	HL	HW	HN
E^1^	0.66*^a^*	0.32*^a^*	0.79*^a^*
Rep (Env)^1^	0.00	0.00	0.00
G**^1^**	3.50*^a^*	0.86*^a^*	1.50*^a^*
G × E^1^	0.60*^a^*	0.31*^a^*	0.53*^a^*
Heritability^2^	0.92	0.84	0.86

Variance components estimate, significance tests, and broad-sense heritability based on the mean values from 2 block replications in 3 field environments. All data from all field environments was used for the analysis presented in the table.

1 G and E indicate genotype and environment, respectively, Rep (Env) indicates replication within environment, and G × E indicates interaction between G and E.

a Significant at *P* ≤ 0.05.

2 Family mean-based broad-sense heritability.

**Fig. 1.**
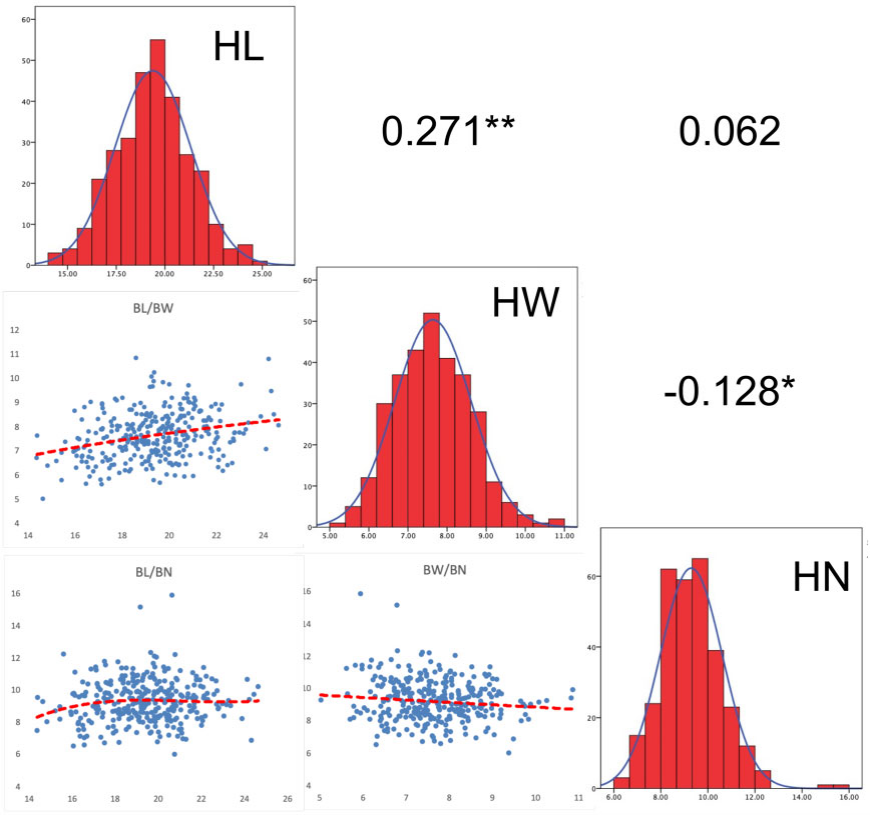
Frequency distributions and correlation of 3 husk traits (BLUP values). Plots on diagonal line show phenotypic distribution of each trait as indicated; values above diagonal line are Pearson’s correlation coefficients between traits; plots below diagonal line are scatter plots of compared traits. *Significant at *P* ≤ 0.05, **Significant at *P* ≤ 0.01.

HL was positively correlated with HW (*r *=* *0.271, *P ≤ *0.01), whereas HW was negatively correlated with HN (*r *=* −*0.128, *P ≤ *0.01); the BLUP values of HL and HN were not correlated (Fig. 1). Similar results were observed in each of single field environment (Supplementary Fig. 1), suggesting that the growth and development of the husk was coordinated with respect to length and width and the number of husk layers had a spatial impact on husk extension.

### The construction of high-density linkage map

**Fig. 2.**
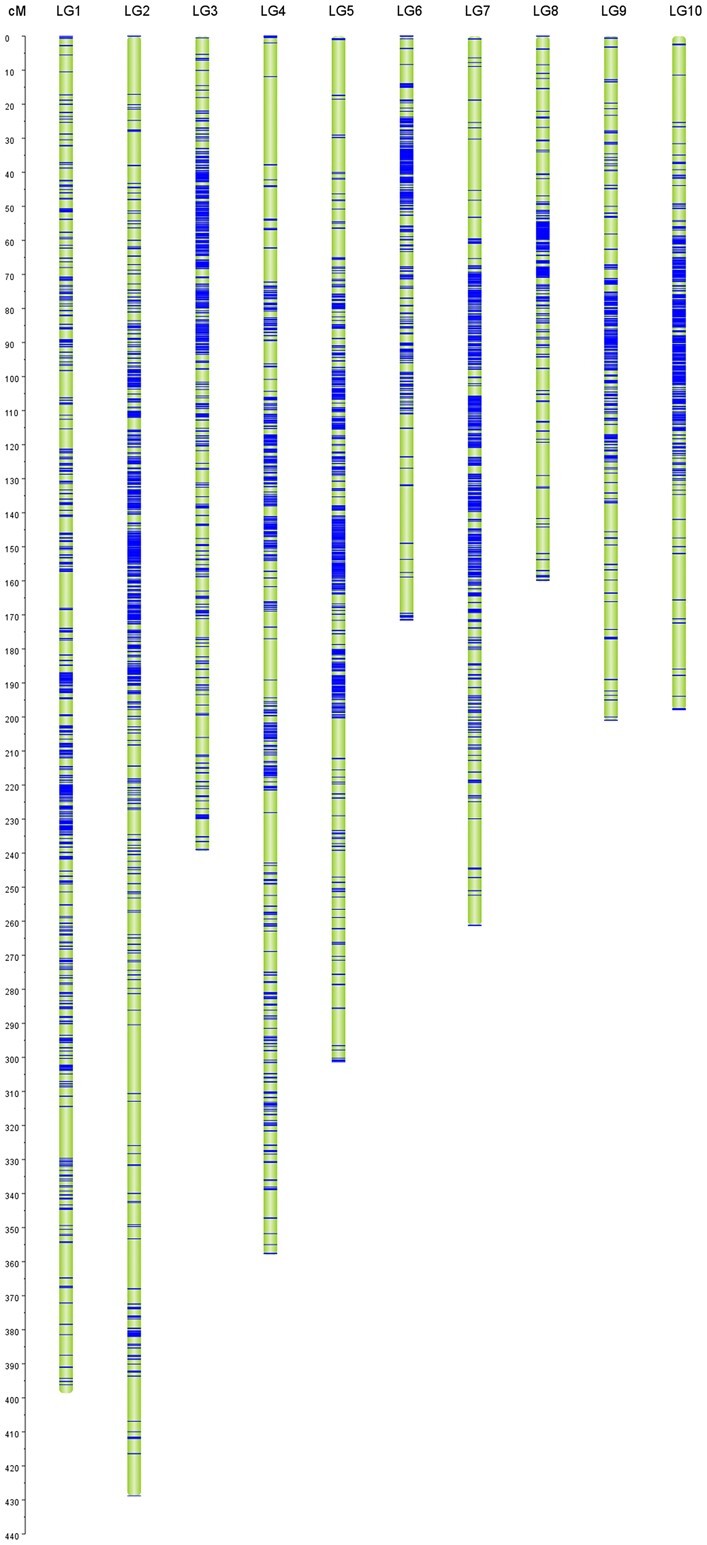
Distribution map of linkage group marker. a) The left scale is relative genetic distance. b) LG 1–10: chromosome number; the horizontal line on the map indicates marker location.

To construct a high-resolution genetic linkage map, 310 RIL individuals and the parental lines was performed using GBS method. In total, 71.336 Gb and 71.662 Gb clean reads were generated for the 2 parents, respectively. A total of 243.3 Gb clean reads were generated for the 310 RIL individuals (Supplementary Table 1). A total of 3,548,904 homozygous SNPs were detected between the 2 parental lines. The SNPs were filtered on the basis of the genotyping criteria, and 8,384 SNPs were retained to generate bin markers among the RIL population. A high-density genetic map was constructed by mapping these 8,384 SNPs into the 10 maize chromosomes (Fig. 2). The total genetic distance of the bin map was 2,718.02 cM, with an average distance of 0.32 cM (Table 3). For chromosome 2, there were 1,136 bin markers covering a genetic length of 428.80 cM, which was the longest genetic length covered among the 10 maize chromosomes. In contract, for chromosome 8 there were 578 bin markers that covered 160.21 cM, the shortest genetic length covered in this map. There were 10 gaps that ranged from 5 to 26 cM in length and the largest gap of 25.89 cM was on chromosome 4.

**Table 3.** Characteristics of the high-density genetic map.

Linkage group (LG)	Number of bin marker	Physical distance (Mb)	Genetic distance (cM)	Average length (cM)	<5 cM gap	Max gap (cM)	***R* ^2^** ^a^
LG01	1018	307.01	398.65	0.39	1009	15.22	0.89
LG02	1136	242.05	428.8	0.38	1123	20.23	0.49
LG03	867	234.82	239.43	0.28	863	6.68	0.81
LG04	941	246.36	358.02	0.38	927	25.89	0.74
LG05	965	223.67	301.36	0.31	956	16.46	0.74
LG06	625	173.87	171.54	0.27	621	16.92	0.91
LG07	882	182.29	261.23	0.3	874	15.08	0.64
LG08	578	180.91	160.21	0.28	570	9.76	0.83
LG09	558	159.71	200.93	0.36	550	11.98	0.84
LG10	814	150.881	197.85	0.24	805	13.91	0.76
Total	8,384	2,101.571	2,718.02	0.32	8,298	25.89	

a *R* ^2^ Determination coefficient between genetic map and physical map.

**Fig. 3.**
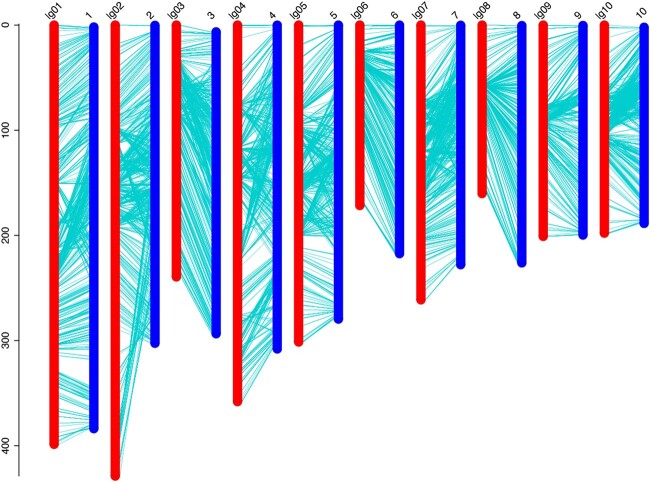
Collinearity analysis between genetic map and physical map. a) The left scale is relative genetic distance. b) 1–10: chromosome number; lg 01–10: linkage group number. c) The left scale (lg 01–10) is linkage group and the right scale (1-10) is chromosome of reference genome maize B73.

To assess the quality and accuracy of this genetic map, the locations of bin markers on the genetic map were compared with the maize B73 RefGen_V4 reference genome (Fig. 3). A high degree of collinearity was observed between the genetic map and the corresponding chromosome. However, there were still few regions displayed inconsistence on several chromosomes. The order of bin markers on the distal ends of chromosomes 2, 4, 5, and 7 was inconsistent with the genetic map (Table 3 and Fig. 3).

### QTL mapping of HL, HW, and HN in each environment

To explore the genetic basis of husk, we performed QTL mapping for HL, HW and HN in 3 field environments and combined environment. QTLs for the 3 husk traits were detected using the CIM method in windows QTL cartographer 2.5. A total of 26 QTLs were identified: 11 QTLs were identified for HL on chromosomes 1, 2, 5, 6, 7, 9, and 10; 8 QTLs for HW on chromosomes 1, 2, 5, and 9; and 7 QTLs for HN on chromosomes 1, 2, 3, 4, 6, and 9 (Table 4, Supplementary Table 2, and Supplementary Figs. 2–4). The confidence intervals for these 26 QTLs spanned physical distances from 0.4 to 16.14 Mb, with an average of 5.42 Mb when compared with the B73 RefGen_v4 genome. The phenotypic variation explained by each QTL ranged from 3.19% to 13.95% of the variation in a trait (Table 4 and Supplementary Table 2).

**Table 4.** QTL identified for HL, HW, and HN in combined environment.

Trait name^a^	QTL name^b^	Chr.	Flanking marker^c^	Interval^d^ (cM)	Physical length^e^ (Mb)	LOD^f^	PVE^g^	ADD^h^
HL	q*HL2-1*	2	mk1520–mk1534	166.14–167.40	1.42	9.32	11.19	0.80
	q*HL5-2*	5	mk4648–mk4675	187.50–194.93	5.09	6.65	6.40	−0.52
	q*HL6*	6	mk5328–mk5332	55.65–56.92	2.75	10.65	11.51	−0.49
	q*HL7-1*	7	mk6267–mk6276	123.71–126.08	15.6	4.86	4.93	−0.42
	q*HL9-2*	9	mk7549–mk7558	130.38–145.60	5.05	3.57	3.89	0.41
	q*HL10*	10	mk8213–mk8233	103.27–107.51	6.06	5.01	5.74	0.50
HW	q*HW1-1*	1	mk844–mk848	286.73–291.16	1.11	3.98	4.83	0.28
	q*HW1-2*	1	mk973–mk981	352.30–358.34	5.30	3.45	5.12	0.23
	q*HW2-1*	2	mk1087–mk1109	76.46–80.99	10.48	4.74	5.70	0.27
	q*HW2-2*	2	mk1112–mk1118	82.05–86.34	10.47	4.89	5.49	0.23
	q*HW5-3*	5	mk4344–mk4383	192.25–192.68	0.89	2.84	3.19	0.21
	q*HW9*	9	mk7086–mk7088	49.50–51.50	0.65	3.70	3.98	0.20
HN	q*HN3-1*	3	mk2187–mk2198	20.06–24.79	6.22	5.52	5.74	0.32
	q*HN4*	4	mk3783–mk3787	256.58–258.06	0.89	9.36	11.1	0.40
	q*HN6*	6	mk5555–mk5564	164.91–166.91	2.14	4.10	5.09	0.30
	q*HN9-1*	9	mk7338–mk7372	91.18–94.23	16.14	9.16	10.09	0.47
	q*HN9-2*	9	mk7428–mk7458	105.18–109.73	6.53	4.06	4.91	0.35

Chr., chromosome.

a Trait is the name of the component of husk: HL, HW, and HN.

b The name of each QTL is a composite of the influenced trait: HL, HW, and HN.

c Flanking markers, the markers to the left and right of the QTL.

d Interval, confidence interval between 2 markers. 1.5-LOD support interval in cM unit.

e Physical length, interval between the 2 markers on the B73 genome.

f LOD, the logarithm of odds score.

g PVE, the phenotypic variance explained by individual QTL.

h ADD, the additive effect value: a positive value indicates that the allele from the female parent (PD80) increased the index of traits, whereas a negative value indicates that the allele from male parent (PHJ65) increased the index of traits.

Twenty QTLs were consistently detected many times. In which, 3 QTLs were identified in 2 field environments, 10 QTLs were identified in both single field environment and combined environment, and 7 QTLs were identified in both multiple field environments and combined environment. Thus, they were viewed as stable QTLs in this study. Nine of the 20 stable QTLs influence HL; 6, HW; and 5, HN. For HL, the QTL on chromosome 6, q*HL6*, was detected in all environments (including 3 field environments and combined environment) and explained 11.51–13.95% of the phenotypic variation, and it had the largest effect of the 9 stable QTLs for that trait. The genetic length of the q*HL6* region was about 1.27 cM, which corresponds to a physical distance of about 2.75 Mb in B73 RefGen_v4. Moreover, q*HL5-2* was detected in the 3 environments, including 2 field environments and combined environment. For HN, q*HN4* was detected in all environments, which explained 10.65–11.66% of the phenotypic variation in HN, had the largest effect of the 5 stable QTLs for the trait. The genetic length of the q*HN4* region was about 1.48 cM, corresponding to a physical distance of about 0.89 Mb.

### Joint mapping for HL, HW, and HN in multiple environments

The phenotype data from 3 field environments and combined environment was used for the Joint mapping. A total of 51 additive QTLs associated with the 3 husk traits were identified. The phenotypic variation explained by each additive QTL ranged from 1.04% to 13.34%: with sums of 94.76%, 47.26%, and 51.17% for HL, HW, and HN, respectively (Supplementary Table 3). Contributions of the interaction between each additive QTL and environment (AE) ranged from 0.92% to 8.23% for HL, HW, and HN. Eighteen additive QTLs for HL were mapped on chromosomes 1, 2, 5, 6, 7, 9, and 10 and included 5 stable QTLs identified using the single-environment analyses. The phenotypic variance explained by each additive QTL was higher than that explained by AE. Thirteen additive QTLs for HW were mapped on chromosomes 1, 2, 4, 6, 7, 8, 9, and 10 and included 4 stable QTLs identified using single-environment mapping. The phenotypic variance explained by each additive QTL was higher than that explained by AE, except for 1 QTL. Twenty additive QTLs for HN were mapped on chromosomes 1, 2, 3, 4, 5, 6, 9, and 10 and included 5 stable QTLs identified using single-environment mapping. The phenotypic variance explained by each additive QTL was higher than that explained by AE.

### Candidate gene prediction

On the basis of the QTL mapping in the RIL population, q*HL*6 and q*HN*4 were inferred to be reliable and major-effect QTLs for HL and HN, respectively. q*HL*6 was mapped between markers mk5328 and mk5332, and it spans a genetic distance of 1.27 cM and corresponds to a physical distance of 2.75 Mb in the B73 RefGen-v4 genome assembly. Through homologous alignment and functional annotation, 7 candidate genes were identified in the QTL region (Table 5). These genes were classified into 2 different functional groups: development process (*Zm00001d052240*, *Zm00001d052245*, and *Zm00001d052254*) and energy metabolism (*Zm00001d052242*, *Zm00001d052243*, *Zm00001d052247*, and *Zm00001d052260*). For q*HN*4, which was mapped between markers mk3783 and mk3787, 19 genes were predicted using the available annotation of B73 RefGen-v4 in the 0.89-Mb target region. These genes were functionally classified into 4 different groups: development progress (*Zm00001d036631*), energy metabolism (*Zm00001d036552*, *Zm00001d036557*, *Zm00001d036564*, *Zm00001d036579*, *Zm00001d036583*, *Zm00001d036608*, and *Zm00001d036608*), metabolic process (*Zm00001d036550*, *Zm00001d036556*, *Zm00001d036570*, *Zm00001d036575*, *Zm00001d036597*, and *Zm00001d036626*), and response to stimulus (*Zm00001d036571*, *Zm00001d036593*, *Zm00001d036613*, *Zm00001d036623*, and *Zm00001d036630*). These results will lay the foundation for analyzing candidate genes related to HL and HN and indicate whether the husk traits are associated with various biological processes.

**Table 5.** Genes located in the intervals of q*HL*4 and q*HN*6.

Gene ID	Chr	Start	End	Description	Biological process
**q*HL*4**					
*Zm00001d052240*	chr4	184,369,041	184,371,794	Pentatricopeptide repeat 5	Development process
*Zm00001d052245*	chr4	184,611,231	184,665,800	Exocyst complex component SEC6 isoform X2	Development process
*Zm00001d052254*	chr4	184,820,400	184,821,661	DNA-binding protein	Development process
*Zm00001d052242*	chr4	184,373,518	184,374,270	ATP synthase delta chain isoform X1	Energy metabolism
*Zm00001d052243*	chr4	184,464,169	184,467,579	Putative laccase precursor	Energy metabolism
*Zm00001d052247*	chr4	18,466,6747	184,669,386	Shikimate kinase	Energy metabolism
*Zm00001d052260*	chr4	185,139,277	185,141,193	Probable galacturonosyltransferase 9	Energy metabolism
**q*HN*6**					
*Zm00001d036631*	chr6	95,649,670	95,655,779	Phosphatidylinositol synthase 2	Development process
*Zm00001d036552*	chr6	91,845,982	91,849,911	Fructose-1,6-bisphosphatase, cytosolic-like	Energy metabolism
*Zm00001d036557*	chr6	92,017,713	92,023,571	External alternative NAD(P)H-ubiquinone oxidoreductase B1, mitochondrial-like isoform X1	Energy metabolism
*Zm00001d036564*	chr6	92,506,962	92,508,996	Thiamine-repressible mitochondrial transport protein THI74-like	Energy metabolism
*Zm00001d036579*	chr6	93,630,607	93,642,846	BEACH domain-containing protein lvsC isoform X1	Energy metabolism
*Zm00001d036583*	chr6	93,694,598	93,724,480	BEACH domain-containing protein lvsC isoform X1	Energy metabolism
*Zm00001d036608*	chr6	94,231,654	94,235,649	Probable alpha-glucosidase Os06g0675700 isoform X2	energy metabolism
*Zm00001d036624*	chr6	95,234,528	95,240,368	Twinkle homolog protein, chloroplastic/ mitochondrial-like	Energy metabolism
*Zm00001d036550*	chr6	91,656,993	91,657,445	Lipid binding protein precursor	Metabolic process
*Zm00001d036556*	chr6	91,870,931	91,874,865	TPA: putative peptidase C48 domain family protein	Metabolic process
*Zm00001d036570*	chr6	93,085,359	93,085,772	60S ribosomal protein L27-3-like	Metabolic process
*Zm00001d036575*	chr6	93,371,896	93,375,751	Putative ribosomal protein S4 (RPS4A) family protein	Metabolic process
*Zm00001d036597*	chr6	94,022,705	94,023,738	sm protein	Metabolic process
*Zm00001d036626*	chr6	95,462,355	95,463,470	Tubulin beta-7 chain-like	Metabolic process
*Zm00001d036571*	chr6	93,093,255	93,103,441	LOW QUALITY PROTEIN: heat shock 70 kDa protein 16-like	Response to stimulus
*Zm00001d036593*	chr6	93,920,665	93,928,357	Auxin response factor 22	Response to stimulus
*Zm00001d036613*	chr6	94,473,770	94,496,176	Probable LRR receptor-like serine/ threonine-protein kinase At3g47570	Response to stimulus
*Zm00001d036623*	chr6	95,057,231	95,057,635	SAUR25—auxin-responsive SAUR family member	Response to stimulus
*Zm00001d036630*	chr6	95,643,471	95,646,716	Filamentation temperature-sensitive H 2B	Response to stimulus

## Discussion

Husk is a lignocellulosic-rich agricultural waste abundantly available throughout the year. Researchers have found that this waste material is profitable from an environmental and economic point of view. For example, husk is used as an animal feed; the fiber, for industrial raw materials; and anthocyanins, for medicine and pigments (Li *et al.* 2008). With machine harvesting, the main economic impact of husk is harvestability. Moisture that evaporates from the kernels must pass through the husk at the dehydration stage (Zhou, Zhang, *et al.* 2016). Unfavorable husk traits, such as too many layers surrounding the ear, limit the speed of dehydration of kernel moisture content at the drying stage. This impedes mechanical harvesting, especially in the northern maize-growing area of China. Thus, understanding the genetic basis of husk traits is beneficial for the genetic improvement of maize husk traits for mechanical harvesting of the kernels (Zhou *et al.* 2020). In this study, a total of 26 QTLs and several candidate genes associated with 3 husk traits, HL, HW, and HH, were detected, which will greatly expand our understanding of the genetic architecture of maize husk.

Furthermore, among the identified QTLs, only 2 QTLs (q*HL*6 and q*HN*4) for HL and HN were insensitive to the 3 field environments; these QTLs were also detected in combined environment, which indicates that QTL-by-environment interaction had a lower effect. This could be because QTLs that explain higher phenotypic variation also have higher direct effects on phenotypes and lower genotype-by-environment interaction effects (Stuber *et al.* 1992). Therefore, information on QTL × environment interaction should be considered carefully, especially for more environment-specific QTLs. These stable and consistent QTLs could be considered priority candidates for molecular marker-assisted selection. In addition, the 3 husk traits in the 3 field environments were found to have wide phenotypic variations with normal distributions, indicating that the heritability was high. The genetic contributions and genetic × environmental interaction effects were also observed to be significant, with positive correlations between HL and HW in the 3 field environments. This indicates an endogenous character, i.e. the growth and development of the husk are coordinated with respect to length and width. In contrast, HN was significantly negatively correlated with HW, supporting the conclusion that the superimposed outgrowth of husk layers could hypothetically limit HL because of the limitation of photosynthetic products (Cui *et al.* 2018).

With the rapid development of high-throughput sequencing technology, various sequencing technologies have been widely used in various research fields (Soon *et al.*, 2013; Wang, Tian, *et al.*, 2020; Wang *et al.*, 2021). GBS is a new and popular method for developing high-density SNPs used for constructing genetic linkage maps, and it has been successfully utilized for genetic studies of complex quantitative traits in maize (Zhou, Zhang, *et al.* 2016; Wang *et al.* 2018; Cui *et al.* 2020). This means that a GBS-based SNP genetic map can detect more recombination events, which would increase the total number of bins and reduce bin size (Zhou, Zhang, *et al.* 2016). In this study, we constructed a genetic map of a maize RIL population derived from PD80 and PHJ65 on the basis of GBS results. The high-density genetic map with 8,384 bin markers was constructed, and it covered 2,718.02 cM with an average marker interval of 0.32 cM. When compared with same population-based genetic maps described in previous studies (Cui *et al.* 2016; Zhou *et al.* 2020), our genetic map covered a similar distance in terms of genome size but had more markers and higher resolution. High-density markers can greatly facilitate the identification of recombinant events and exact recombinant breakpoints, which significantly affects the accuracy of QTL mapping. Increasing the density of markers distributed around the entire genome improves the resolution of genetic maps (Zou *et al.* 2012).

We identified a new series of QTLs and pinpointed candidate genes associated with the husk traits. Twenty-nine candidate genes were identified in the region of q*HL*6 and q*HN*4, of which 17 candidate genes potentially play a role in biological processes related to metabolism. Previous studies have shown that metabolism is an indispensable part of a plant life cycle and contributes to a large part of plant phenotype performance (Hunter *et al.* 2012; Xiao *et al.* 2016). Some metabolic pathways influence plant growth and development (Kramer and Ackelsberg 2015). Therefore, it is not surprising to find that a large number of candidate genes involved in metabolism are associated with HL and number because husk growth is a dynamic process that involves an interconnected series of metabolic pathways. Our findings can be used as a useful resource for performing functional studies aimed at understanding the molecular pathways involved in husk growth and development.

## Supplementary Material

jkac198_Figure_legends

jkac198_Supplementary_Figure_S1

jkac198_Supplementary_Figure_S2

jkac198_Supplementary_Figure_S3

jkac198_Supplementary_Figure_S4

jkac198_Supplementary_Table_S1

jkac198_Supplementary_Table_S2

jkac198_Supplementary_Table_S3

## Data Availability

The data underlying this article are available at https://doi.org/10.6084/m9.figshare.19369520.v2, including the RIL phenotypic data for statistical and QTL analyses, genotypic scores, and linkage map. The data underlying this article are available in the Supplementary material. Distributions of the phenotypic data in the RIL population are shown in Supplementary Fig. 1. Supplementary Fig. 2 shows the distribution of QTL for HL across the entire genome in the RIL population. Supplementary Fig. 3 indicates the distribution of QTL for HW across the entire genome in the RIL population. Supplementary Fig. 4 shows the distribution of QTL for husk number across the entire genome in the RIL population. Supplementary Table 1 represents the statistics of sequencing data. Supplementary Table 2 shows the QTL identified for HL, HW, and HN in 3 field environments. Supplementary Table 3 shows the additive QTL and their environment interaction effects for HL, HW, and HN in 3 field environments and combined environment. Supplemental material is available at *G3* online.
