# Supplementary material for: Molecular mapping of quantitative trait loci for 3 husk traits using genotyping by sequencing in maize (Zea mays L.)
Source: G3 (Bethesda). 2022 Aug 9;12(10):jkac198. doi: 10.1093/g3journal/jkac198 (PMC9526056; doi:10.1093/g3journal/jkac198)
Supplement: jkac198_Figure_legends [file jkac198_figure_legends.docx]

**Figure legends**

**Figure S1**: Frequency distributions and correlation of three husk traits in three environments. **Figure S2**: Distribution of QTL for husk length across the entire genome in the RIL population. **Figure S3**: Distribution of QTL for husk width across the entire genome in the RIL population. **Figure S4**: Distribution of QTL for husk number across the entire genome in the RIL population.
