# Supplementary figures and images for: Molecular mapping of quantitative trait loci for 3 husk traits using genotyping by sequencing in maize (Zea mays L.)
Source: G3 (Bethesda). 2022 Aug 9;12(10):jkac198. doi: 10.1093/g3journal/jkac198 (PMC9526056; doi:10.1093/g3journal/jkac198)

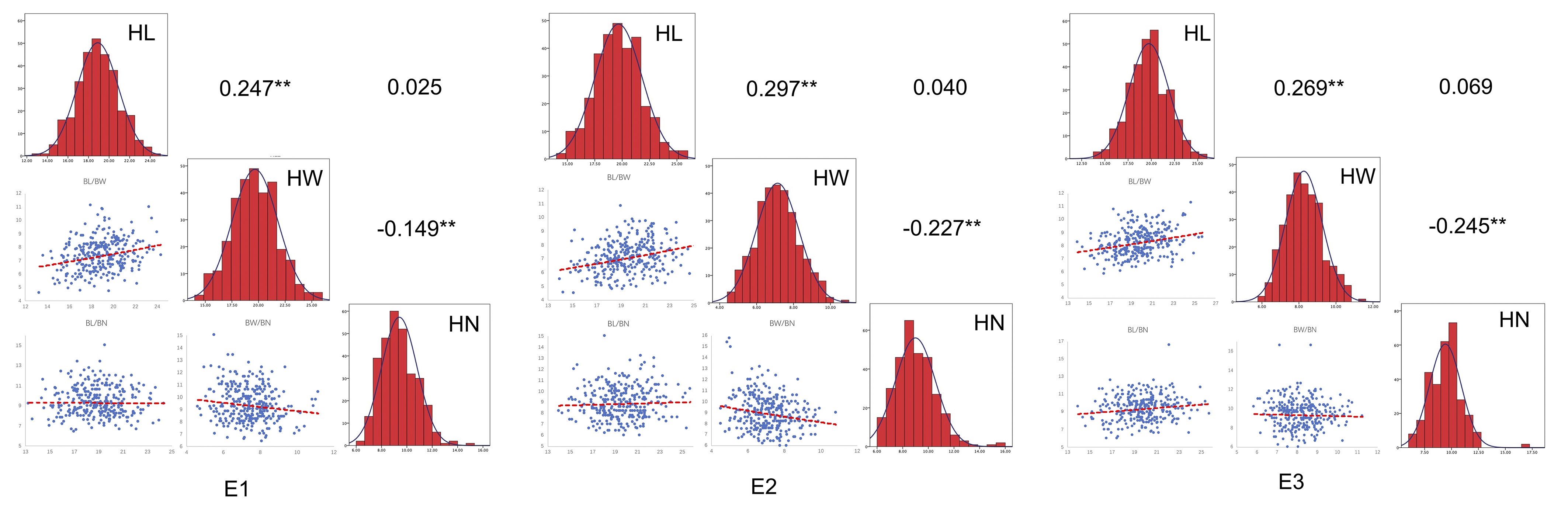

Supplement: jkac198_Supplementary_Figure_S1 [file jkac198_supplementary_figure_s1.jpeg]

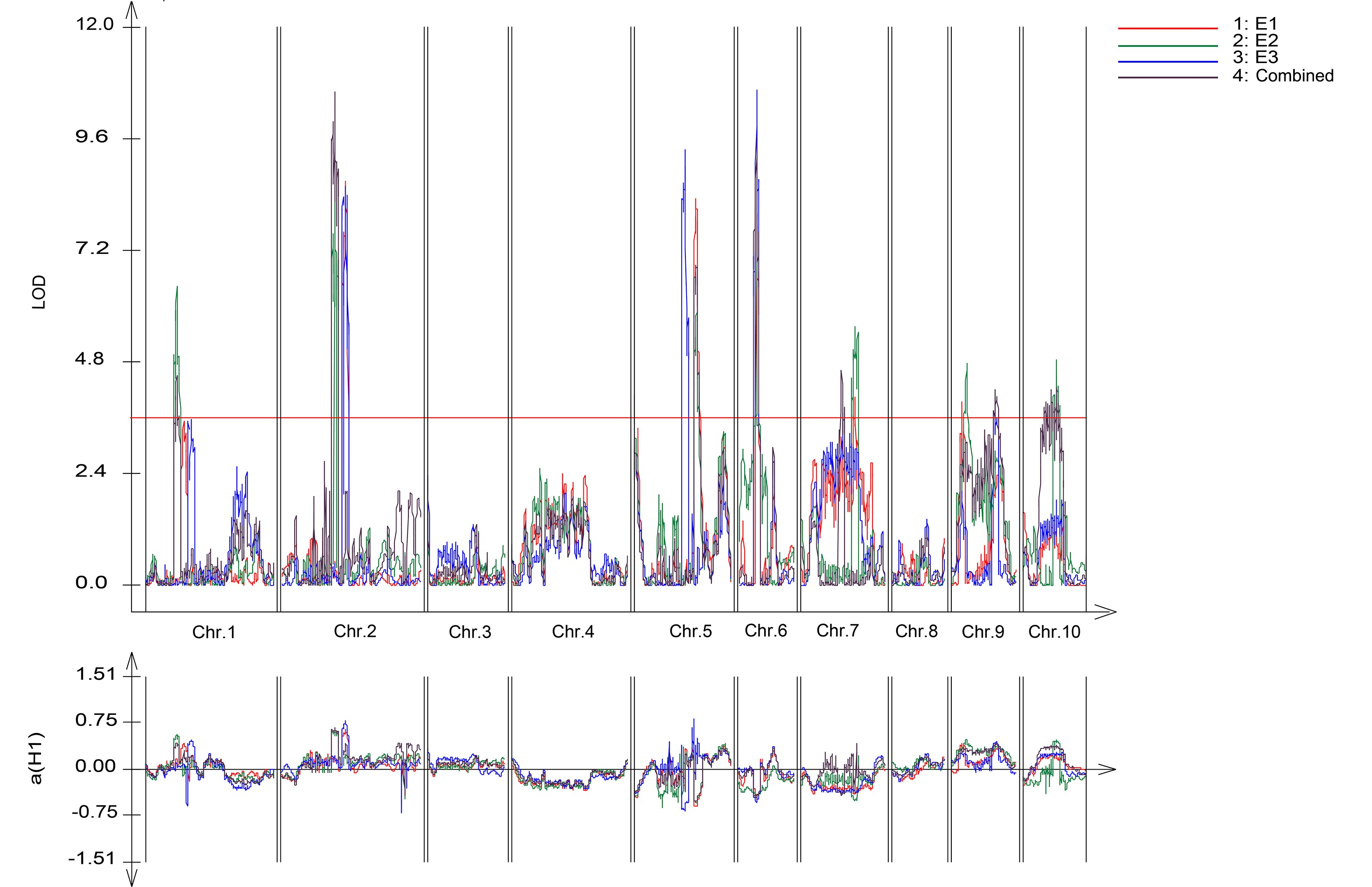

Supplement: jkac198_Supplementary_Figure_S2 [file jkac198_supplementary_figure_s2.jpeg]

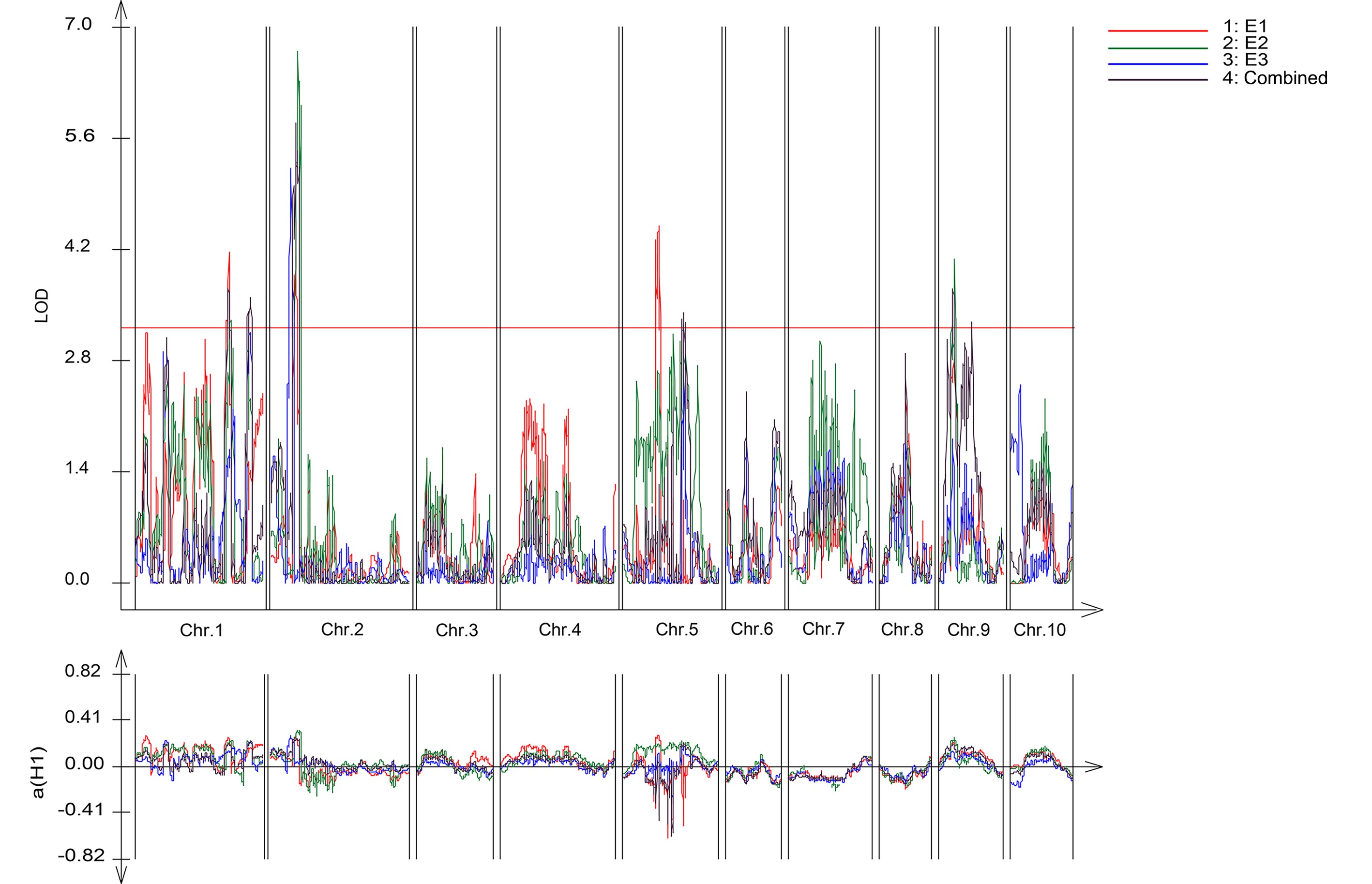

Supplement: jkac198_Supplementary_Figure_S3 [file jkac198_supplementary_figure_s3.jpeg]

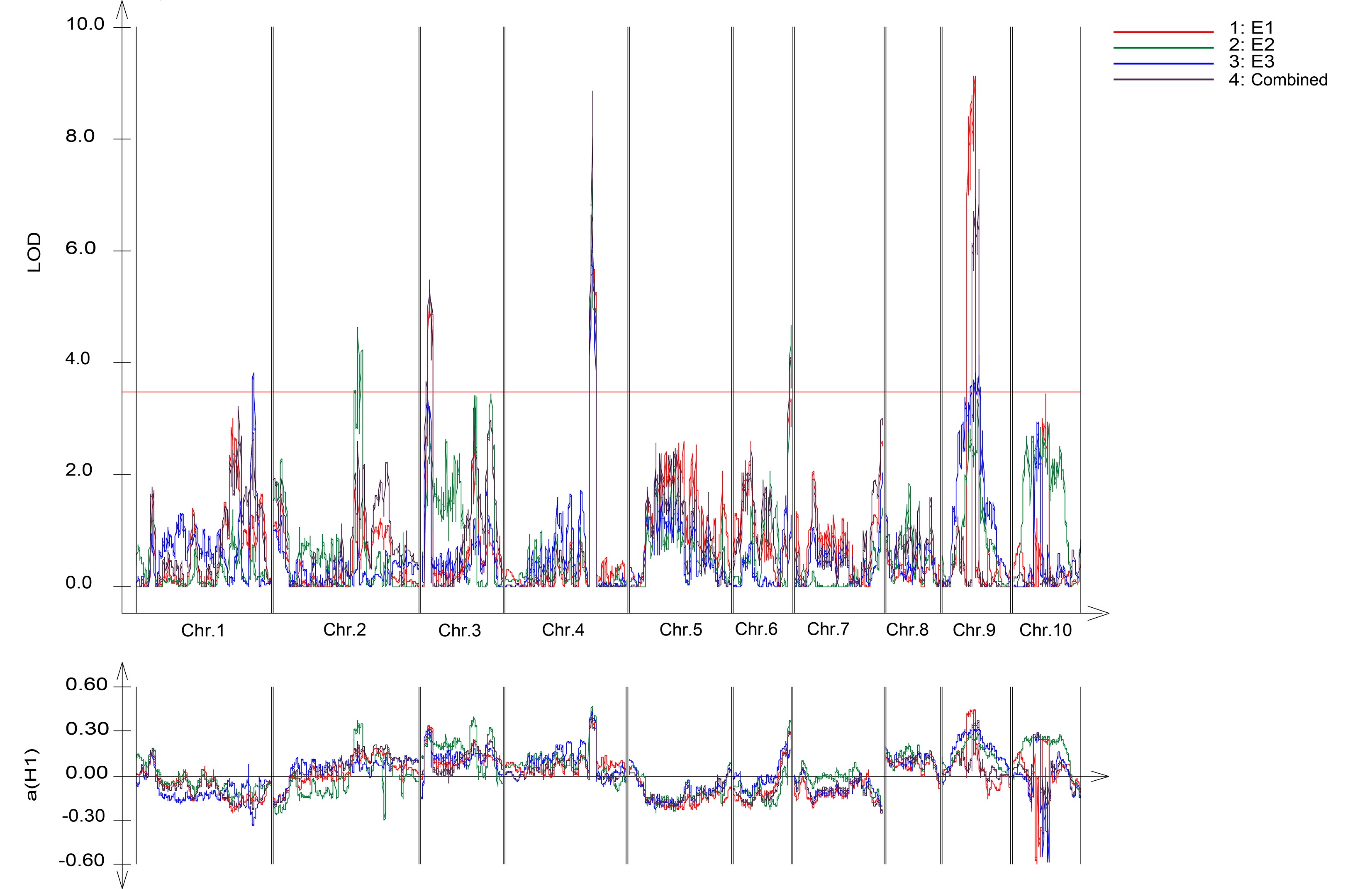

Supplement: jkac198_Supplementary_Figure_S4 [file jkac198_supplementary_figure_s4.jpeg]
