# Supplementary material for: Molecular mapping of quantitative trait loci for 3 husk traits using genotyping by sequencing in maize (Zea mays L.)
Source: G3 (Bethesda). 2022 Aug 9;12(10):jkac198. doi: 10.1093/g3journal/jkac198 (PMC9526056; doi:10.1093/g3journal/jkac198)
Supplement: jkac198_Supplementary_Table_S2 [file jkac198_supplementary_table_s2.docx]

**Table S2. QTL identified for HL, HW and HN in three field environments.**

| Trait  name^a^ | QTL  Name^b^ | Environments^c^ | Chr.^d^ | Flanking marker | Interval^f^(cM) | Physical length^g^ (Mb) | LOD^h^ | PVE^i^ | ADD^j^ |
| --- | --- | --- | --- | --- | --- | --- | --- | --- | --- |
| HL | qHL1 | E1 | 1 | mk174-mk232 | 116.38-120.38 | 13.4 | 3.76 | 4.54 | 0.43 |
|  | qHL2-2 |  | 2 | mk1981-mk1994 | 197.20-200.61 | 5.31 | 8.96 | 10.13 | 0.6 |
|  | qHL5-2 |  | 5 | mk4648-mk4675 | 187.50-194.93 | 5.09 | 5.32 | 5.92 | -0.6 |
|  | qHL6 |  | 6 | mk5328-mk5332 | 55.65-56.92 | 2.75 | 11.33 | 13.95 | -0.59 |
|  | qHL7-1 |  | 7 | mk6267-mk6276 | 123.71-126.08 | 15.6 | 4.25 | 4.12 | -0.44 |
|  | qHL7-2 |  | 7 | mk6254-mk6270 | 163.34-168.46 | 4.37 | 6.21 | 7.1 | -0.66 |
|  | qHL9-1 |  | 9 | mk7082-mk7086 | 41.58-51.00 | 2.43 | 4.85 | 5.16 | 0.51 |
|  | qHL2-1 | E2 | 2 | mk1520-mk1534 | 166.14-167.40 | 1.42 | 9.11 | 10.79 | 0.7 |
|  | qHL5-1 |  | 5 | mk4086-mk4110 | 86.78-89.91 | 9.33 | 7.6 | 8.41 | -0.64 |
|  | qHL5-2 |  | 5 | mk4648-mk4675 | 187.50-195.10 | 5.09 | 5.65 | 6.14 | -0.54 |
|  | qHL6 |  | 6 | mk5328-mk5332 | 55.65-56.92 | 2.75 | 10.74 | 11.64 | -0.48 |
|  | qHL7-2 |  | 7 | mk6254-mk6270 | 163.34-168.46 | 4.37 | 6.09 | 7.02 | -0.49 |
|  | qHL9-1 |  | 9 | mk7082-mk7086 | 41.58-51.00 | 2.43 | 4.45 | 5.21 | 0.49 |
|  | qHL10 |  | 10 | mk8213-mk8233 | 103.27-107.51 | 6.06 | 5.21 | 5.96 | 0.54 |
|  | qHL2-2 | E3 | 2 | mk1981-mk1986 | 197.20-199.84 | 4.75 | 8.48 | 9.93 | 0.68 |
|  | qHL6 |  | 6 | mk5328-mk5332 | 55.65-56.92 | 2.75 | 10.91 | 12.35 | -0.57 |
|  | qHL9-2 |  | 9 | mk7549-mk7558 | 130.38-145.60 | 5.05 | 3.98 | 4.24 | 0.45 |
| HW | qHW1-1 | E1 | 1 | mk844-mk848 | 286.73-291.16 | 1.11 | 4.68 | 5.32 | 0.26 |
|  | qHW2-1 |  | 2 | mk1087-mk1109 | 76.46-80.99 | 10.48 | 4.38 | 5.28 | 0.29 |
|  | qHW5-1 |  | 5 | mk4501-mk4530 | 110.11-113.65 | 1.3 | 4.5 | 5.32 | 0.27 |
|  | qHW9 |  | 9 | mk7086-mk7088 | 49.50-51.50 | 0.65 | 3.56 | 3.56 | 0.22 |
|  | qHW1-1 | E2 | 1 | mk844-mk848 | 286.73-291.16 | 1.11 | 3.84 | 4.91 | 0.24 |
|  | qHW2-2 |  | 2 | mk1112-mk1118 | 82.05-86.34 | 10.47 | 6.72 | 8.18 | 0.29 |
|  | qHW5-2 |  | 5 | mk4715-mk4719 | 159.84-160.11 | 0.4 | 3.14 | 3.49 | 0.22 |
|  | qHW9 |  | 9 | mk7088-mk7088 | 51.00-51.50 | 0.45 | 3.39 | 3.22 | 0.25 |
|  | qHW1-2 | E3 | 1 | mk980-mk981 | 354.27-358.34 | 3.99 | 3.76 | 5.08 | 0.24 |
|  | qHW5-3 |  | 5 | mk4344-mk4383 | 192.25-192.68 | 0.89 | 3.21 | 3.4 | 0.17 |
| HN | qHN3 | E1 | 3 | mk2187-mk2198 | 20.06-24.79 | 6.22 | 5.24 | 5.41 | 0.33 |
|  | qHN4 |  | 4 | mk3783-mk3787 | 256.58-258.06 | 0.89 | 9.73 | 11.66 | 0.6 |
|  | qHN6 |  | 6 | mk5555-mk5564 | 164.91-166.91 | 2.14 | 5.35 | 5.4 | 0.39 |
|  | qHN9-1 |  | 9 | mk7338-mk7372 | 91.18-94.23 | 16.14 | 9.11 | 10.02 | 0.44 |
|  | qHN2 | E2 | 2 | mk2100-mk2108 | 245.86-249.99 | 0.81 | 4.63 | 5.46 | 0.37 |
|  | qHN4 |  | 4 | mk3783-mk3787 | 256.58-259.35 | 0.89 | 9.24 | 10.98 | 0.59 |
|  | qHN6 |  | 6 | mk5555-mk5564 | 164.91-166.91 | 2.03 | 4.54 | 5.11 | 0.36 |
|  | qHN1 | E3 | 1 | mk963-mk970 | 343.34-349.40 | 5.47 | 3.82 | 4.98 | -0.33 |
|  | qHN3 |  | 3 | mk2187-mk2198 | 20.06-24.79 | 6.22 | 5.67 | 6.07 | 0.32 |
|  | qHN4 |  | 4 | mk3783-mk3787 | 256.58-258.06 | 0.89 | 9.12 | 10.65 | 0.57 |
|  | qHN9-2 |  | 9 | mk7428-mk7458 | 105.18-109.73 | 6.53 | 4.2 | 4.82 | 0.31 |

^a^Trait is the name of the component of husk: *HL* husk length, *HW* husk width, *HN* husk number.

^b^The name of each QTL is a composite of the influenced trait: HL, HW, HN.

^c^E1: Zhoukou; E2: Yuanyang; E3: Anyang;

^d^Chr. Chromosome.

^e^Flanking markers, the markers to the left and right of the QTL.

^f^Interval, confidence interval between two markers. 1.5-LOD support interval in cM unit.

^g^Physical length, interval between the two markers on the B73 genome.

^h^LOD, the logarithm of odds score.

^i^PVE, the phenotypic variance explained by individual QTL.

^j^ADD, the additive effect value. A positive value indicates that the allele from the female parent (PD80) increased the index of traits, whereas a negative value indicates that the allele from male parent (PHJ65) increased the index of traits.
