# Supplementary material for: Molecular mapping of quantitative trait loci for 3 husk traits using genotyping by sequencing in maize (Zea mays L.)
Source: G3 (Bethesda). 2022 Aug 9;12(10):jkac198. doi: 10.1093/g3journal/jkac198 (PMC9526056; doi:10.1093/g3journal/jkac198)
Supplement: jkac198_Supplementary_Table_S3 [file jkac198_supplementary_table_s3.docx]

**Table S2.** Additive QTL and their environment interaction effects for HL, HW and HN in three field environments and BLUPs.

| **Trait^a^** | **Chr.^b^** | **Flanking markers^c^** | **Interval^d^ (cM)** | **LOD^e^** | **PVE(A)^f^**  **(%)** | **PVE(AE)^g^**  **(%)** | **Add^h^** | **Interaction effect between additive QTL and environment** | | | |
| --- | --- | --- | --- | --- | --- | --- | --- | --- | --- | --- | --- |
|  |  |  |  |  |  |  |  | **AE1** | **AE2** | **AE3** | **AE4** |
| HL | 1 | mk208-mk210 | 93.5-94.5 | 8.53 | 3.38 | 1.92 | 0.17 | -0.10 | -0.09 | -0.08 | 0.26 |
|  | 1 | mk225-mk215 | 131.5-134.5 | 8.29 | 4.44 | 1.38 | 0.15 | -0.10 | 0.38 | -0.13 | -0.15 |
|  | 1 | mk841-mk832 | 282.5-283.5 | 6.61 | 3.21 | 2.19 | -0.19 | 0.16 | 0.04 | -0.20 | 0.00 |
|  | 2 | mk1160-mk1161 | 89.5-91.5 | 17.91 | 8.57 | 3.03 | 0.22 | 0.51 | -0.19 | -0.14 | -0.18 |
|  | 2 | mk1175-mk1265 | 131.5-132.5 | 10.37 | 5.66 | 1.67 | 0.16 | -0.16 | 0.43 | -0.11 | -0.16 |
|  | 2 | mk1983-mk1985 | 197.5-199.5 | 14.73 | 8.00 | 4.45 | 0.27 | -0.12 | -0.15 | 0.41 | -0.14 |
|  | 5 | mk4674-mk4263 | 158.5-159.5 | 9.32 | 5.13 | 1.58 | -0.16 | 0.15 | 0.14 | -0.41 | 0.13 |
|  | 5 | mk4383-mk4643 | 192.5-193.5 | 18.25 | 8.11 | 3.65 | -0.24 | 0.25 | -0.21 | 0.28 | -0.32 |
|  | 5 | mk4922-mk4925 | 272.5-275.5 | 9.93 | 4.27 | 2.95 | 0.22 | -0.15 | -0.13 | 0.11 | 0.18 |
|  | 5 | mk4935-mk4937 | 285.5-287.5 | 8.48 | 3.90 | 2.82 | 0.21 | 0.13 | 0.12 | -0.17 | -0.08 |
|  | 6 | mk5329-mk5330 | 55.5-56.5 | 24.07 | 11.65 | 8.23 | -0.36 | -0.05 | 0.36 | -0.29 | -0.02 |
|  | 6 | mk5512-mk5513 | 105.5-107.5 | 9.22 | 3.66 | 1.37 | 0.15 | -0.15 | -0.03 | -0.14 | 0.32 |
|  | 6 | mk5539-mk5545 | 125.5-128.5 | 10.08 | 4.58 | 3.81 | -0.25 | 0.11 | 0.10 | -0.14 | -0.08 |
|  | 7 | mk6254-mk6264 | 162.5-163.5 | 14.19 | 5.72 | 3.23 | -0.23 | -0.16 | 0.19 | 0.20 | -0.24 |
|  | 9 | mk7072-mk7073 | 31.5-32.5 | 8.88 | 3.78 | 3.44 | 0.23 | 0.08 | -0.12 | 0.00 | 0.04 |
|  | 9 | mk7085-mk7086 | 44.5-50.5 | 6.33 | 3.18 | 2.03 | 0.18 | -0.14 | 0.21 | 0.02 | -0.10 |
|  | 9 | mk7558-mk7559 | 145.5-147.5 | 10.89 | 4.78 | 2.56 | 0.20 | 0.32 | -0.14 | -0.13 | -0.05 |
|  | 10 | mk7897-mk8043 | 87.5-88.5 | 6.15 | 2.75 | 1.32 | 0.14 | 0.22 | -0.15 | 0.06 | -0.13 |
| HW | 1 | mk96-mk100 | 65.5-67.5 | 6.37 | 2.63 | 1.62 | 0.11 | 0.13 | -0.05 | -0.09 | 0.01 |
|  | 1 | mk433-mk630 | 150.5-151.5 | 10.13 | 4.20 | 2.91 | 0.14 | -0.13 | 0.10 | -0.05 | 0.08 |
|  | 1 | mk874-mk883 | 300.5-302.5 | 10.52 | 4.51 | 3.59 | 0.16 | -0.12 | 0.10 | -0.02 | 0.04 |
|  | 1 | mk992-mk994 | 378.5-384.5 | 5.59 | 2.39 | 1.69 | 0.11 | 0.10 | 0.02 | -0.10 | -0.02 |
|  | 2 | mk1073-mk1075 | 61.5-62.5 | 5.52 | 2.37 | 1.20 | 0.09 | -0.06 | -0.04 | 0.16 | -0.05 |
|  | 2 | mk1109-mk1111 | 79.5-80.5 | 14.99 | 6.01 | 3.84 | 0.16 | 0.14 | -0.13 | -0.12 | 0.11 |
|  | 2 | mk1135-mk1115 | 85.5-86.5 | 7.85 | 4.08 | 1.15 | 0.09 | -0.12 | 0.24 | -0.05 | -0.07 |
|  | 4 | mk3287-mk3299 | 83.5-84.5 | 5.69 | 2.37 | 1.88 | 0.11 | 0.09 | -0.03 | -0.07 | 0.00 |
|  | 6 | mk5541-mk5546 | 141.5-151.5 | 5.97 | 2.44 | 2.44 | -0.13 | 0.01 | 0.00 | -0.01 | -0.01 |
|  | 7 | mk5864-mk5949 | 148.5-149.5 | 5.45 | 2.64 | 1.56 | -0.10 | 0.04 | -0.13 | -0.01 | 0.11 |
|  | 8 | mk6936-mk6938 | 79.5-81.5 | 10.86 | 4.45 | 4.36 | -0.18 | -0.04 | 0.02 | 0.02 | -0.01 |
|  | 9 | mk7086-mk7088 | 50.5-51.5 | 16.22 | 7.10 | 6.30 | 0.21 | 0.00 | 0.09 | -0.12 | 0.02 |
|  | 10 | mk8100-mk8123 | 95.5-97.5 | 5.06 | 2.06 | 2.00 | 0.12 | 0.01 | 0.01 | -0.03 | 0.01 |
| HN | 1 | mk57-mk55 | 42.5-44.5 | 5.28 | 1.38 | 1.28 | 0.15 | -0.05 | 0.06 | 0.02 | -0.03 |
|  | 1 | mk58-mk62 | 45.5-50.5 | 5.77 | 1.46 | 1.39 | 0.16 | -0.04 | 0.06 | 0.00 | -0.03 |
|  | 1 | mk895-mk896 | 302.5-303.5 | 6.67 | 1.49 | 1.26 | -0.15 | 0.11 | -0.04 | -0.01 | -0.06 |
|  | 1 | mk969-mk970 | 344.5-348.5 | 7.21 | 2.24 | 1.56 | -0.17 | 0.10 | 0.00 | -0.18 | 0.09 |
|  | 2 | mk1033-mk1034 | 20.5-23.5 | 10.39 | 2.25 | 2.21 | -0.20 | 0.02 | -0.01 | 0.03 | -0.04 |
|  | 2 | mk2087-mk2089 | 237.5-239.5 | 5.24 | 1.29 | 1.06 | 0.14 | 0.09 | 0.01 | -0.02 | -0.09 |
|  | 2 | mk2101-mk2107 | 246.5-248.5 | 6.94 | 1.26 | 0.90 | 0.13 | -0.11 | -0.02 | 0.02 | 0.11 |
|  | 3 | mk2190-mk2193 | 23.5-24.5 | 16.71 | 3.37 | 2.56 | 0.21 | 0.08 | 0.01 | -0.20 | 0.10 |
|  | 3 | mk2217-mk2219 | 29.5-30.5 | 6.09 | 1.99 | 1.07 | 0.14 | -0.09 | 0.06 | 0.18 | -0.15 |
|  | 3 | mk2869-mk2872 | 151.5-153.5 | 7.03 | 2.38 | 1.10 | 0.14 | -0.12 | 0.25 | -0.01 | -0.12 |
|  | 3 | mk2964-mk2965 | 199.5-203.5 | 6.09 | 1.37 | 1.28 | 0.15 | -0.06 | 0.04 | -0.02 | 0.03 |
|  | 4 | mk3707-mk3737 | 220.5-225.5 | 5.17 | 1.22 | 1.17 | 0.14 | 0.00 | -0.03 | 0.04 | -0.02 |
|  | 4 | mk3786-mk3785 | 257.5-258.5 | 30.22 | 6.99 | 5.23 | 0.31 | 0.05 | 0.15 | -0.30 | 0.11 |
|  | 5 | mk4086-mk4109 | 79.5-80.5 | 5.29 | 1.41 | 1.30 | -0.15 | 0.00 | -0.07 | 0.00 | 0.06 |
|  | 5 | mk4093-mk4097 | 131.5-133.5 | 5.06 | 1.35 | 1.25 | -0.15 | 0.02 | -0.06 | -0.01 | 0.05 |
|  | 6 | mk5324-mk5328 | 52.5-55.5 | 7.01 | 1.58 | 1.54 | -0.17 | -0.01 | -0.03 | 0.04 | 0.00 |
|  | 6 | mk5541-mk5546 | 145.5-148.5 | 7.70 | 1.77 | 1.39 | 0.16 | 0.13 | -0.08 | 0.00 | -0.06 |
|  | 6 | mk5555-mk5564 | 164.5-169.5 | 6.29 | 2.00 | 1.21 | 0.15 | -0.04 | 0.18 | 0.01 | -0.15 |
|  | 9 | mk7085-mk7086 | 44.5-47.5 | 5.47 | 1.04 | 0.92 | 0.13 | -0.01 | -0.06 | 0.00 | 0.07 |
|  | 9 | mk7153-mk7144 | 81.5-82.5 | 6.29 | 0.99 | 0.35 | 0.08 | -0.08 | -0.07 | -0.04 | 0.18 |
|  | 10 | mk8082-mk7708 | 95.5-96.5 | 44.43 | 13.34 | 3.68 | 0.26 | 0.72 | -0.24 | -0.27 | -0.21 |
|  | 10 | mk8306-mk8305 | 113.5-114.5 | 5.80 | 0.92 | 0.30 | 0.07 | -0.08 | -0.03 | -0.07 | 0.18 |

^a^Trait, is the name of the component of husk: *HL* husk length, *HW* husk width, *HN* husk number.

^b^Chr. Chromosome.

^c^Flanking markers, the markers to the left and right of the QTL.

^d^Interval, confidence interval between two markers.

^e^LOD, the logarithm of odds score.

^f^PVE, the phenotypic variance explained by individual QTL.

^g^ADD, the additive effect value: A positive value indicates that the allele from the female parent (PD80) increased the index of traits, whereas a negative value indicates that the allele from male parent (PHJ65) increased the index of traits.

^h^AE, interaction between additive effect and environment.

AE1-4, interaction between additive effect and Zhoukou, Yuanyang, Anyang and BLUP, respectively.
